# Supplementary figures and images for: The miR-221/222 cluster regulates hematopoietic stem cell quiescence and multipotency by suppressing both Fos/AP-1/IEG pathway activation and stress-like differentiation to granulocytes
Source: PLoS Biol. 2023 Nov 20;21(11):e3002015. doi: 10.1371/journal.pbio.3002015 (PMC10695376; doi:10.1371/journal.pbio.3002015)

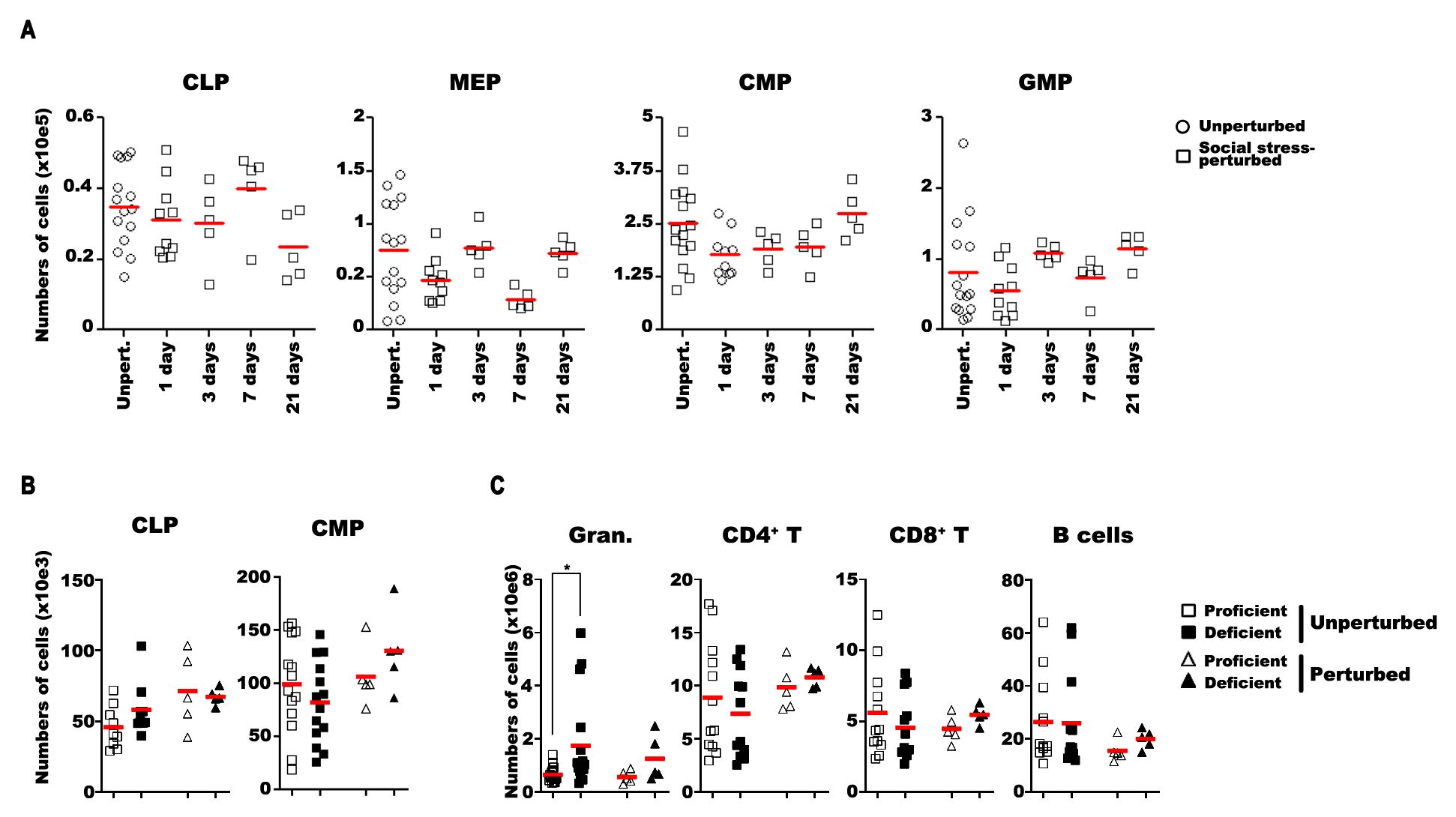

Supplement: S1 Fig — (A) Single-cell suspensions of 2 tibia and femurs of miR-221/222-proficient unperturbed mice or of 1 day, 3 days, 7 days, or 21 days after social stress–perturbed mice or (B) of miR-221/222-proficient and miR-221/222-deficient unperturbed or of 1 day after social stress–perturbed mice or (C) from spleens of miR-221/222-proficient and miR-221/222-deficient unperturbed or of 1 day after social stress–perturbed mice were prepared in matched pairs, analyzed with flow cytometry, and the numbers of cells were plotted. Numbers of different cell populations from unperturbed miR-221/222-proficient (open squares) and miR-221/222-deficient (closed squares) mice or perturbed miR-221/222-proficient (open triangles) or miR-221/222-deficient (closed triangle) are presented. Red lines indicate the mean values. One-way ANOVA with Tukey posttest was used to evaluate statistical significance (* indicates p < 0.05. The numerical data can be found in S1 Data and on FlowRepository.org through FR-FCM-Z6PS accession number). (TIF) [file pbio.3002015.s004.tif]

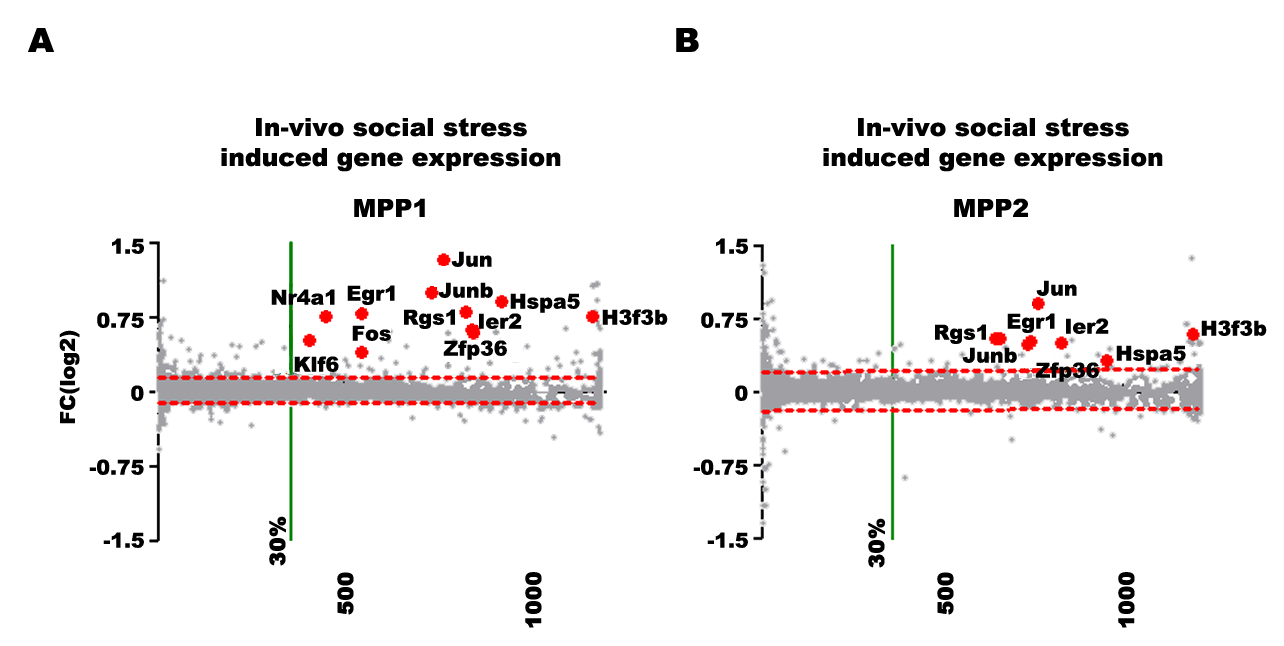

Supplement: S2 Fig — Genes with higher expression after short-term perturbation (red dots and gene symbols are selected genes) in (A) MPP1 and (B) MPP2 cells are presented by comparative differential expression analysis of unperturbed versus perturbed cells. Differentially expressed genes are above the significance limit (red dashed). The log2 fold-change expression values were plotted against the numbers of unperturbed cells expressing the gene. The right side of the green line indicates genes expressed in more than 30% of the cells. The numerical data can be found in S2 Data. (TIF) [file pbio.3002015.s005.tif]

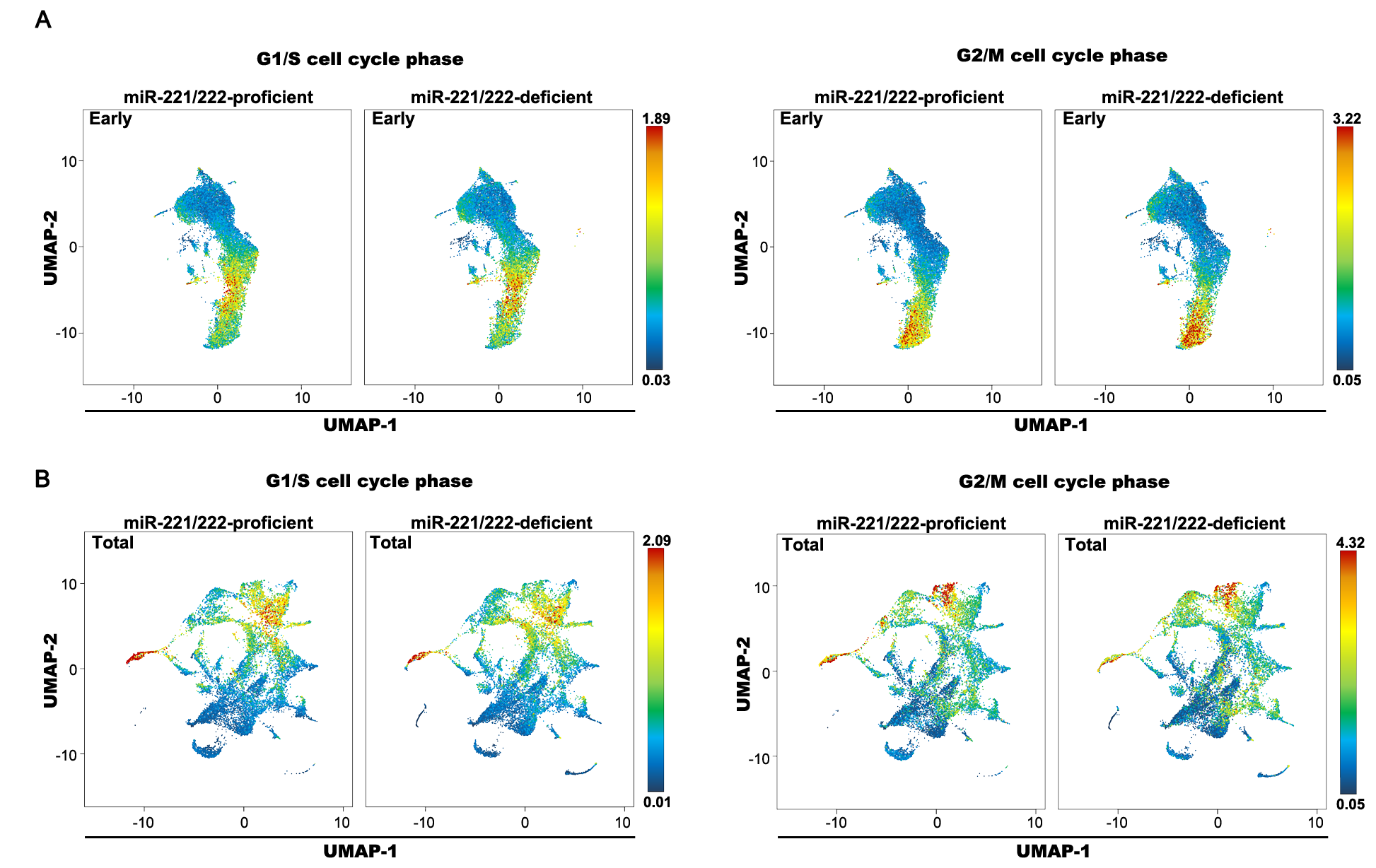

Supplement: S3 Fig — (A) The integrated data of miR-221/222-proficient and miR-221/222-deficient unperturbed, short-term perturbed, and serial transplanted HSC, MPP1, and MPP2 populations after single-cell transcriptome sequencing (early) or (B) HSC, MPP1, MPP2, MPP, CLP, lin−ckit+Sca1− and lin−ckit−Sca1− populations (total) were further analyzed by a UMAP. Cells with characteristic gene expression pattern for G1/S (left) or G2/M (right) cell cycle–related genes are shown on a composite picture for proficient and for deficient cells. For plotting, gene-set modules of G1/S and G2/M genes were used. The numerical data can be found in S2 Data. CLP, common lymphoid progenitor; HSC, hematopoietic stem cell; MPP, multipotent progenitor; UMAP, Uniform Manifold Approximation and Projection for Dimension Reduction. (TIF) [file pbio.3002015.s006.tif]

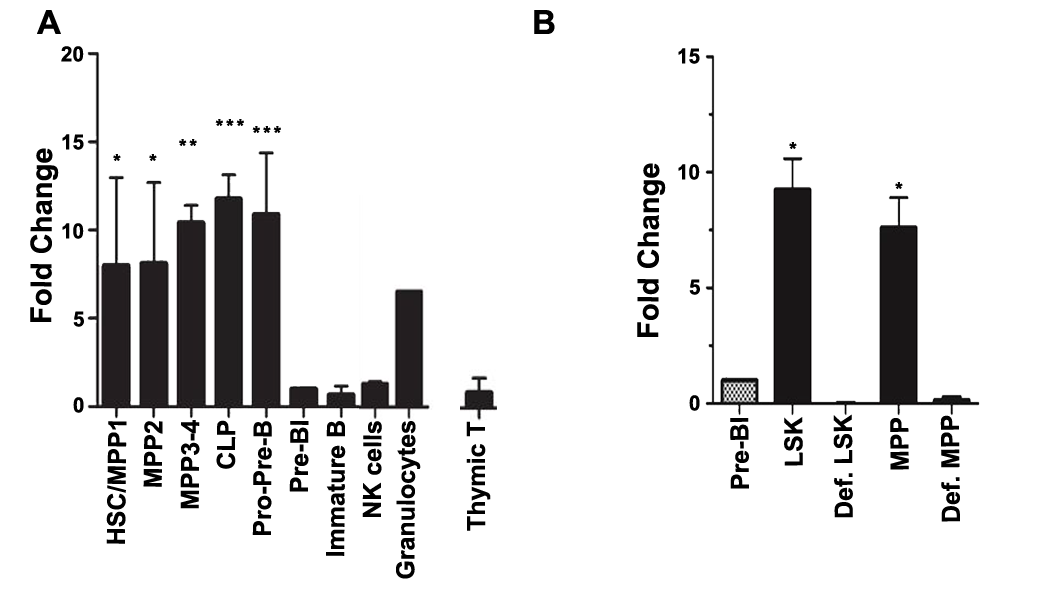

Supplement: S4 Fig — (A) Relative miR-221 expression in bulk sorted hematopoietic progenitor, B cell subsets, NK cells, granulocytes, and T cells in BM and thymus (n = 5 mice). Expression of miR-221 in the different bulk sorted populations are displayed as fold change relative to miR-221 expression in pre-BI cells. (B) Relative miR-221 expression in BM of miRNA-proficient and miRNA-deficient LSK and MPP subsets (n = 5 mice). One-way ANOVA with Dunnett posttest was used to evaluate statistical significance (*, **, and ***, indicate p < 0.05, p < 0.01, and p < 0.001, respectively). HSC/MPP1: pool of hematopoietic stem cell and multipotent progenitor (MPP)1, MPP3-4: pool of MPP3 and MPP4 populations, CLP: common lymphoid progenitor, Pre-BI: precursor BI cell, Pro-Pre-B: progenitor of precursor B cell, NK cell: natural killer cells. LSK: BM-derived lin−Kit+Sca1+ population. The numerical data for Fig 1B–1F can be found in S1 Data. BM, bone marrow; HSC, hematopoietic stem cell; miRNA, microRNA; MPP, multipotent progenitor; NK, natural killer. (TIF) [file pbio.3002015.s007.tif]

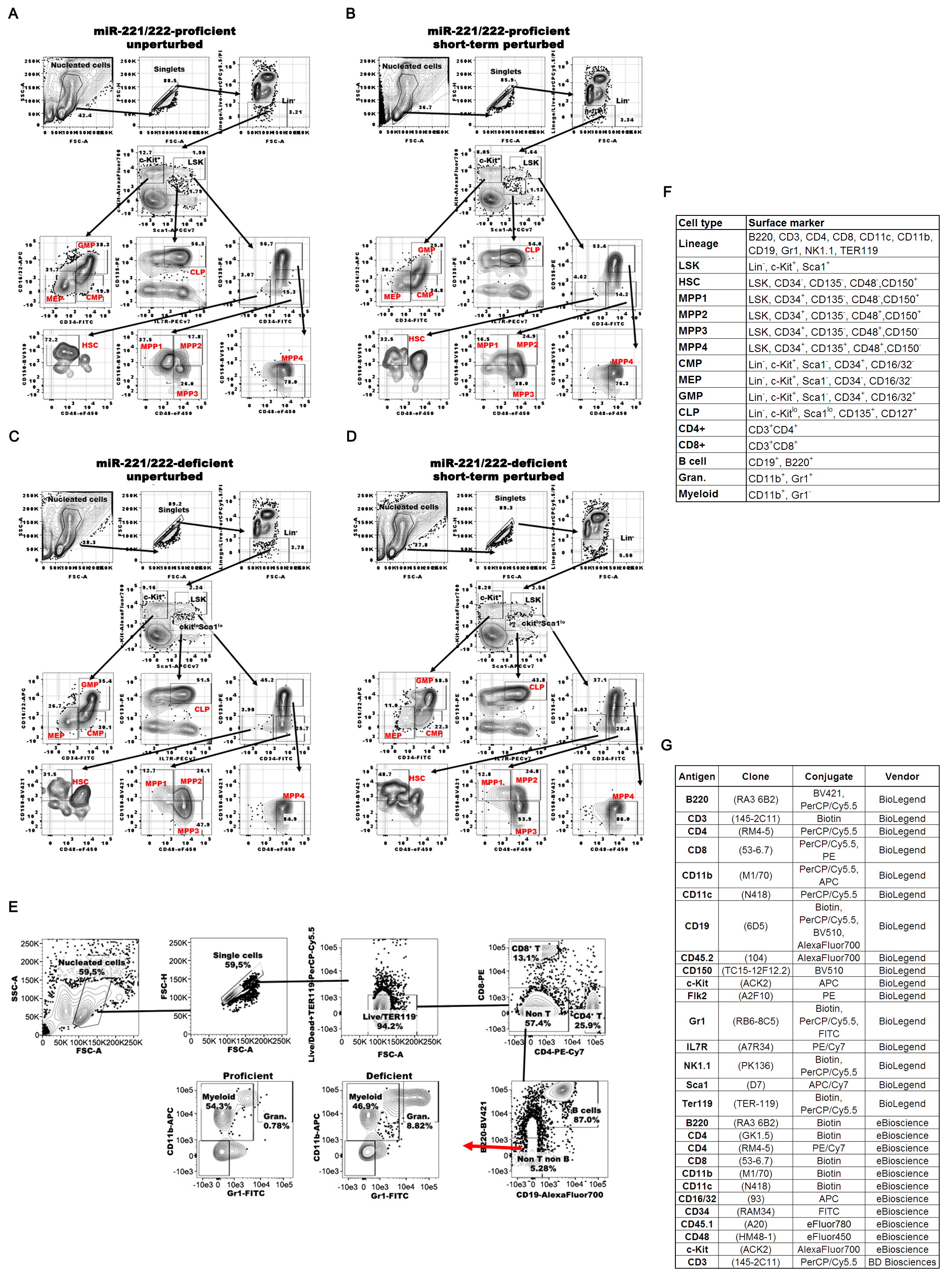

Supplement: S5 Fig — After preparing single cell suspensions of (A) unperturbed or (B) perturbed miR-221/222-proficient or of (C) unperturbed or (D) perturbed miR-221/222-deficient BM, stem and progenitor cells were stained for flow cytometry analysis. (E) Single-cell suspension of miR-221/222-proficient or miR-221/222-deficient spleen or blood was prepared, and the major hematopoietic lineage cells were stained for flow cytometry analysis. (F) Surface marker expression of the measured hematopoietic stem, progenitor, and lineage cells. HSC were gated on lin− (B220−CD3−CD4−CD8−CD19−CD11c−CD11b−Gr1−NK1.1−TER119−) c-kit+Sca1+Flk2−CD34−CD150+CD48− cells. MPP1 was gated on lin−c-kit+Sca1+Flk2−CD34+CD150+CD48− cells. MPP2 were gated on lin−c-kit+Sca1+Flk2−CD34+CD150+CD48+ cells. MPP3 were gated on lin−c-kit+Sca1+Flk2−CD34+CD150−CD48+ cells. MPP4 were gated on lin−c-kit+Sca1+Flk2+CD34+CD150−CD48+ cells. CLPs were gated on lin−c-kitloSca1loFlk2+IL7R+ cells. CMPs were gated on lin−c-kit+Sca1−CD34+CD16/32− cells. MEPs were gated on lin−c-kit+Sca1−CD34−CD16/32− cells. GMPs were gated on lin−c-kit+Sca1−CD34+CD16/32+ cells. CD4 T cells were gated as CD3+CD4+; CD8 T cells were gated as CD3+CD8+ cell. B cells were gated as CD4−CD8−B220+CD19+ cell. Myeloid cells were gated as CD4−CD8−B220−CD19−CD11b+Gr1− cells. Granulocytes (Gran.) were gated on CD4−CD8−B220−CD19−CD11b+Gr1+ cells. (G) Antibodies used in the analyses. The numerical data can be found in the FlowRepository.org through FR-FCM-Z6PS accession number. BM, bone marrow; CLP, common lymphoid progenitor; CMP, common myeloid progenitor; GMP, granulocyte-myelocyte progenitor; HSC, hematopoietic stem cell; MEP, megakaryocyte-erythroid progenitor; MPP, multipotent progenitor. (TIF) [file pbio.3002015.s008.tif]

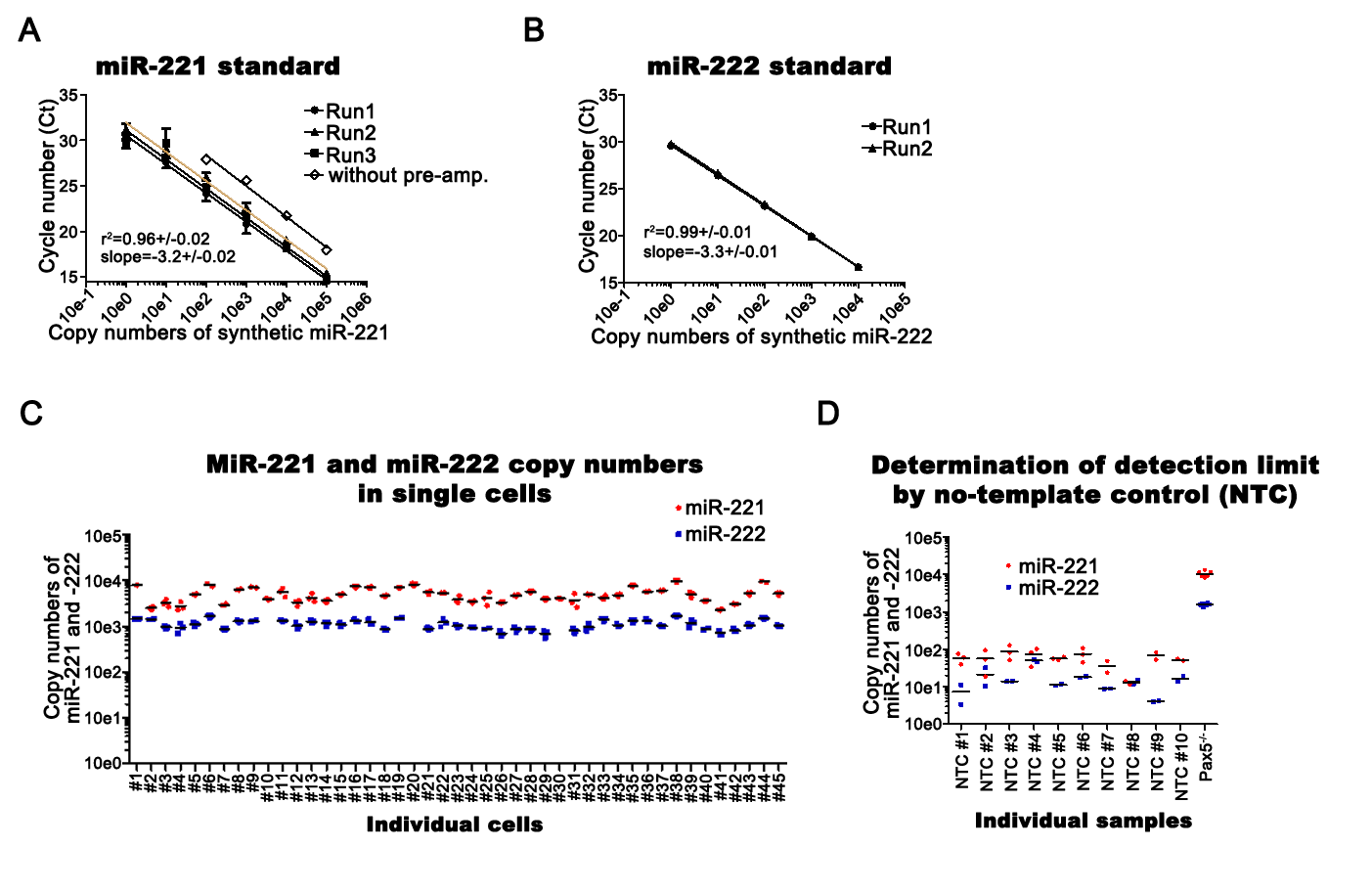

Supplement: S6 Fig — (A) Serial dilution of 105-100 copies of synthetic miR-221 oligonucleotide was measured 3 times (Run1-3) in 3 technical replicates. The TaqMan qPCR measurements were done after or without (without pre-amp) 10-cycle PRC amplification of the reverse transcript. (B) Serial dilution of 105-100 copies of synthetic miR-222 oligonucleotides were measured 2 times (Run1 and 2) in 3 technical replicates. The TaqMan qPCR measurements were done after 10-cycle PRC amplification of the reverse transcript. (C) A total of 45 single-cell sorted miR-221/222-proficient lin−c-kit+Sca1+CD150+CD48− (pool of HSC and MPP1) cells were directly sorted in lysis buffer, suitable for miR-221- or miR-222-specific reverse transcription reaction. After 10-cycle PRC amplification of the reverse transcript, the copy numbers of miR-221 (red) and miR-222 (blue) were determined in individual cells using standard curves developed in the same reaction plate. Red and blue dots indicate technical replicates (D) The limit of detection in miR-221 and -222 copy numbers were assigned by the highest value of 10 NTCs, where no cell was sorted in the lysis buffer. Red and blue dots indicate technical replicates. Pax5−/− pre-BI cells were used as positive control. The mean of the technical replicates are calculated and plotted for 10 individual cells. The numerical data can be found in S1 Data. HSC, hematopoietic stem cell; MPP, multipotent progenitor; NTC, no template control. (TIF) [file pbio.3002015.s009.tif]
