# Supplementary material for: The miR-221/222 cluster regulates hematopoietic stem cell quiescence and multipotency by suppressing both Fos/AP-1/IEG pathway activation and stress-like differentiation to granulocytes
Source: PLoS Biol. 2023 Nov 20;21(11):e3002015. doi: 10.1371/journal.pbio.3002015 (PMC10695376; doi:10.1371/journal.pbio.3002015)
Supplement: Attachment — Submitted filename: Response to Reviewers.docx [file pbio.3002015.s010.docx]

**Point-by-point replies to the Reviewers' comments**

**Reviewer 1:**

Jani et al show that miR-221/222 deletion in hematopoietic stem cells (using Vav-iCre) affects the gene expression program of HSC and early multipotent progenitors in unperturbed and perturbed states (i.e. social disturbance), and that this results in changes in the number of precursors and mature granulocytes. Competitive serial transplantation revealed a substantial defect in miR-221/222 deficient cells in at least one set of experiments. The paper contains a distracting section about "ex vivo/in vitro stress" that is not important for the overall conclusions of the paper and may have a serious flaw in data interpretation. The handling of the direct effects of miR-221/222 on target mRNAs may reveal a lack of miRNA expertise on the research team. This part should be revisited. Nevertheless, the biological effects, if reproduced, are very interesting and worthy of report.

1. The first section of the results ("Controlled perturbations of steady-state hematopoiesis can be induced..) is redundant with a long section in the introduction, and references no new data or figure panels. One of these sections should be eliminated.

We thank the Reviewer for drawing our attention to this unnecessary repetition. We have deleted the repetitive parts from the results section, revised the introduction, and we have also rewritten the abstract and parts of the discussion, as also requested by Reviewer 2 and 3.

2. The "ex vivo/in vitro" stress results are distracting and unnecessary, especially because of one major caveat to their interpretation.

The higher abundance of IEG genes in cells allowed to continue transcription during processing may reflect increased transcription due to activation of signaling pathways during processing, as the authors suggest. However, it is also possible that these genes are expressed at a constant basal rate in vivo and during processing, and that the observed differences in transcript abundance at the end of processing with or without ActD treatment reflects the very short half life of most of these mRNAs. That is, they are degraded during processing, and can't be replaced in the presence of ActD. Many of the highlighted IEGs are known to have very short-lived mRNAs, and ActD treatment of cells is the standard way to reveal this.

This possibility is not mutually exclusive with transcriptional activation, but it is important to distinguish these effects. Is there really a transcriptional stress response going on during processing? Or is there a technical artefact in the gene expression analysis that is uncovered by ActD treatment? A more controlled in vitro stress would be preferable to this passive protocol that depends on ActD. It would also help to see evidence that stress pathways are induced (as in ref 11), assuming signaling state may be as easy to measure before and after processing.

Another reasonable course of action would be to remove this section from the paper and just state that ActD was used during processing to avoid any effect of transcription occurring during cell processing.

We thank the Reviewer for his “in depth” considerations of interpreting transcript pools of genes in unperturbed and perturbed (socially stressed) HSC, MPP1 and MPP2. We take his suggested reasonable course of action and have now removed the section of the paper describing the differential single cell RNAseq analyses in the presence and absence od ActD (**Fig 1B**). As suggested by the Reviewer, we now just state in the Materials and Methods and in the results that ActD was used during cell isolation and processing to avoid transcription occurring during cell preparation and processing.

3. Line 234-235: "[miR221/222] is turned off in natural killer (NK), thymocytes and T lymphocytes, and preB and B lymphocytes." This should more specifically reference the data in Supplementary Fig. 4A, which is focused on bone marrow and thymus. Some peripheral B and T cells do express miR-221/222, where it has been shown to function in immune responses.

As suggested by the Reviewer, we now reference the data in **Supplementary Fig 4A**. We have also modified the manuscript to add two references (refs in the modified ms.) describing increased miR-221/222 expression upon activation in peripheral T or B cells in mice.

4. The Methods section is inadequate in several sections, because the supplementary methods were not provided in the pdf package for reviewers. In particular, the single cell quantification of miRNA expression is not explained at all, though it seems quite novel and interesting. I see little need for supplementary methods, and recommend putting all relevant information in the main manuscript.

We have now incorporated all information previously found in Supplementary Methods into the Method section, which means we have deleted the Supplementary Method.

5. The insertion of figure legends within the Results section is distracting. Also, Figure 2 legend is repeated.

We agree with the Reviewer, that the insertion of the Figure captions within the main text, separated from the figure, is distracting. However, in doing so, we have followed the guidelines of PLOS Biology for manuscript preparation (https://journals.plos.org/plosbiology/s/figures#loc-how-to-submit-figures-and-captions). According to the Reviewer’s suggestion we have now attempted to separate the Figure captions embedded in the main text in a more visible way. Also, we have removed one of the captions for **Fig 2**.

6. Lines 269-274: miR-221/222 is on the X chromosome, not the Y chromosome.

We thank Reviewers 1 and 2 for pointing out this mistake. We are embarrassed not detecting this mistake in our manuscript. Of course we know, that miR221/222 is on the X chromosome, a knowledge which we use in breeding the miR221/222-deficient mice used in our experiments. We have corrected the mistake throughout the manuscript.

7. The direct targets of miR-221/222 would not be expected to have large individual changes. Finding one that was increased 8.5 fold (Fos) is not good experimental evidence of direct miRNA targeting of the Fos mRNA, but likely reflects secondary effects on Fos (like several other IEGs, as observed by the authors). These secondary effects are of interest(!), but they should be characterized as the emergent phenotypic effect of miR-221/222 deficiency, and potential direct targets should be assessed as well.

To detect direct miR-221/222 silencing activity, the gene expression data should be analyzed using gene set analyses, or CDF plots that compare the entire catalog of expressed miR-221/222 predicted targets with all other genes as a group. The shift in expression of this group of genes might be as little as 5% or as much as 30% for a high abundance miRNA. This kind of analysis should be done for HSC, MPP1 and MPP2. This sentence about miR-221/222 targeets in the Discussion (lines 562-565) is badly off track. Fos was not shown to be a target in this study. Other targets were not shown to be unaffected. A proper analysis of miR-221/222 requirements for target gene repression could and should be provided, even if individual targets are not functionally linked to the observed emergent phenotypes of miR-221/222 deficiency.

We found experimental evidence by M. C. Errico et al. (1) validating Fos as direct target of miR‑221/222. These literature data which we reference now in the manuscript and our experimental results allowed us to suggest that Fos is a direct miR-221/222 target in hematopoietic stem cells, while the other differentially upregulated genes are indirectly regulated by miR-221/222. We have attempted to identify genes with increased expression in miRNA-221/222-deficient HSC, MPP1 and MPP2 as a consequence of direct or indirect miRNA-dependent action on gene expression. We agree with the Reviewer, that we have not validated the differentially upregulated transcription of Fos gene as a consequence of missing molecular interaction between miR‑221/222 and Fos mRNA in miRNA deficient cells by standard molecular biology experiments. Since neither HSC, nor MPP1 nor MPP2 can be kept for sufficient length of time in tissue culture as stably differentiated cells, and since one mouse provides at best between 5000 and 10 000 of these cells, proper validation experiments with the right cells are practically impossible to do.

Moreover, when we have done full-length mRNA sequencing analysis on HSC and MPP1 cells, we found in the 3’-UTR of Fos mRNA the experimentally validated miR-221/222 seed sequence. If wanted these sequencing data can be provided.

We have changed the wording of our conclusions from our findings, that Fos appears differentially upregulated in miR-221/222-deficient, unperturbed HSC and in miR-221/222-deficient perturbed HSC, MPP1 and MPP2. We agree, that our differential gene expression analyses only allow to suggest, that such changes in mRNA pools are the result of differential miR-221/222 action. We now word our conclusions to say, that this result SUGGESTS an upregulated pool of Fos transcripts, which, in turn, SUGGESTS an upregulation of Fos/Jun AP-1 pools, which, in turn, SUGGEST an upregulation of the AP-1-controlled transcription of the indirect targets, most of them immediate early genes.

We provide the data of the requested analysis on our scRNA-seq data, in which we made gene set enrichment analyses (GSEA) using predicted or experimentally validated miRNA‑221/222 target gene expression (**Fig 1A**) in our unperturbed HSC, MPP1 and MPP2 dataset (the method is in greater details described below in the caption of **Fig 1**). These analyses show that the target gene expression is almost equally distributed without significant accumulation in any E-cluster between HSC, MPP1 and MPP2 cells (**Fig 1B**). We also could not detect significant difference in the cumulative expression between miR‑221/222-proficient and deficient cells, in analyses the Reviewer suggested (**Fig 1C**). Additionally we plotted the differential expression of each of the predicted target genes against the frequency of cells expressing the gene. We found two-to-three genes having marginal (~5%) higher expression in at least 10% of deficient cells (**Fig1D-F**). Worth noting, that two-to-three genes were also found to be higher expressed at marginal level (~5%) in miR-221/222-proficien cells indicating a stochastic differential expression value around the 5% boundary. We found only Fos as experimentally validated direct target expressed ~20% higher in more than 60% of miR‑221/222-deficient HSC and MPP1 cells.

**Fig 1: Gene set enrichment analysis and differential gene expression analysis of predicted miR‑221/222 target genes in scRNA-seq data of miR-221/222-proficient and deficient HSC, MPP1 and MPP2 cells.** (**A)** Selection of 310 predicted miR-221/222 target genes identified by at least two of three bioinformatical miRNA target prediction tools (TarBase v8, TargetScan Release 8.0 and miRDB). (**B**) GSEA was performed for each cell based on the difference to the mean of log normalized expression values of all cells in the analysed set as pre-ranked list and 1000 randomizations (2, 3). Significant up- or downregulation was defined by a FDR ≤ 0.50 and normalized p-value < 0.05. For visualization, normalized expression score (NES) for significant cells were plotted. The GSEA was performed for indicated cells using the 310 predicted miR-221/222 target genes. (**C**) Violin plots show the mean (red bars) and median (black bars) differences of the NES of the GSEA in miR-221/222-proficeient and deficient HSC, MPP1 and MPP2 cells. Significant differences between the NES values were calculated by Mann-Whitney U-test. (**D-F**) Differential expression analyses of predicted miR-221/222 target gene expressions in miR-221/222-proficient and deficient HSC (**D**), MPP1 (**E**) and MPP2 (**F**). The red dashed line indicates the 5% differential expression band.

8. The results in Figure 7 are striking. Are they reproducible in independent experiments? It appears that all replication shown in the figure are from recipients of the same transferred HSCs. An experiment of this importance bears repeating.

We agree with the Reviewer, that such a striking change in the properties of transplanted HSC should reflect to the properties of HSC of each donor mouse. A repetition of such an extensive experiment of serial transplantations would take at least 8-9 months, far exceeding the time-frame for the revision. This estimation of the required time does not take into consideration the time it might take to get the permissions from the controlling authorities of Berlin to actually repeat this experiment.

In our first, single (not serial) transplantation experiments, not included in the submitted paper, we used LT-HCS cells, i.e. LSK CD150^+^CD48^-^. Already after 3 months, miR‑221/222-deficient cells showed reduced lymphoid repopulation capacities. Following Cabezas-Wallscheid´s et al. (4) findings, that LT-HSC are mixtures of HSC and MPP1, and that only HSC have long-term repopulation capacities, and taking our own findings in account, that miR‑221/222-deficiency reduced the numbers of HSC 2.5-fold (see **Fig 2C** of the manuscript), while increasing the numbers of MPP1, we used the same number (100) of deficient and proficient HSC for our serial transplantation experiments shown in **Fig 7**. We also transplanted 100 MPP1 cells and we got comparable results as Cabezas-Wallscheid´s et al. (4) indicating that MPP1 cells are not capable of hematopoietic reconstitution. These data are not shown in the manuscript.

In order to avoid the limitation of not having biological variances of FACS enriched HSCs, we pooled 3 donor mice for the first, and 5 or 6 donor mice for the second HSC transplantations. This diminished the possibility that a single HSC donor would bias the readout of the experiment. The numbers of animals in the analyses (5-6), all of the deficient cells showing the same reduction of lymphoid regeneration capacities, and all of them showing equally high repopulation capacities of the HSC and MPP compartments in bone marrow (**Fig 7C**), are considered as independent biological parallel measurer of miR-221/222-proficiency or -deficiency. We have now extended the methods describing the serial transplantation analyses in greater details.

**Reviewer 2:**

Jani et al present a novel mechanism for miRNA-221/222 in regulating the early progenitor compartment of the hematopoietic system- as well as providing important information about transcriptional alterations associated with isolation / purification stress on early progenitor cells. They evaluate the changes in number as well as the shifts in transcriptional profiles in stem and early progenitors after various stress and point to miRNA-221/222 as key players for the prevention of activation of these early primitive cells. They utilize a model where miRNA-221/222 is conditionally ablated in Vav-expressing cells (hematopoietic cells). While not critical to the findings, as the crosses still will ablate the miRNA 221/222 expression, it is a bit concerning that the authors have described this as a locus on the Y chromosome, but it appears to be located on the X chromosome. There are interesting and relevant data regarding stress and activation of early progenitors cells that would be relevant the stem cell community.

Major issues

1) The authors have presented this as a sex specific phenomenon (with several references to the miRNA 221/222 locus being on the Y chromosome. The crosses do work (regardless of if the locus is on the X or Y chromosome), but do clarify the location of the locus. As this is located on a sex chromosome the authors should examine if female mice progenitor cells respond differently to the stress (relevant with either X or Y location)

As we have done already in replying to Reviewer 1, we are embarrassed for not detecting this mistake. We have corrected the error in the text of the manuscript.

We are aware of the differences in hematopoiesis influenced by (sex)hormones, however these differences also in context with stress-related responses were not the primary interest of the current manuscript, therefore all experiments were done in male mice to exclude possible gender related differences. We acknowledge that hematopoiesis in response to social stress in different genders and in different age might be regulated differently by miR-221/222 and it is an interesting field for discovery.

2) The stress response of the HSCs reported, looks similar to the "activated" state of stem cells reported by Cabezas-Wallscheid (doi.org/10.1016/j.cell.2017.04.018) and the authors should compare the scRNA profiles generated with this data set-

Cabezas-Wallscheid describe the transition from inactive, dormant HSCs (stage 1) to active HSCs (stage 4). Similarly to these results, we also found that E-cluster 0 resting HSC is in gradual connection to cell cycle entering E-cluster 1. Our analyses find this cell cycle-directed pathway of activation not directly connected with IEG expressing E-cluster 2 in unperturbed hematopoiesis (See **Fig 1C,D,F** of the manuscript). Thus, when we compared with GSEA the scRNA-seq transcriptional data which derived from activated/stage 4 HSC cells with the transcriptional data derived after social stress perturbation, we found that the two activation-related transcriptional programs are significantly different. In short, the development of IEG-expressing activated cells are missing in the analyses of Cabezas-Wallscheid et al.. The results of these analyses is summarized below on **Fig 2**.

Since there is no unified and standardized method for scRNA-seq data processing, one should take caution when comparing datasets generated by different single cell RNA-seq technologies. Different sequencing depth, sequencing background determinations and exclusion parameters, row data normalization methods and the different numbers of cells involved in the analysis can cause alterations in result read-out. Since we have no explanation for these differences, we kindly ask the reviewer for the permission, not to include the results of these analyses in our manuscript. As we present them here, these analyses could be included as supplementary information, without the need to discuss them.

**Fig 2: Comparison of HSC activation programs.** (**A**) In primary cluster analysis of the transcriptomes of unperturbed and socially perturbed HSC, MPP1 and MPP2, seven E-clusters (E for early hematopoiesis) were identified. (The panel is taken from **Fig 1C** of the manuscript.) (**B**) We performed a differential expression analysis between dormant/stage 1 and activated/stage 4 using Mann-Whitney U test. Significantly differentially expressed genes were defined by the adjusted *p* value below 0.05 and logFC of 0.25. Stage 1 and Stage 4 representative gene sets were defend by significantly differentially upregulated genes in stage 1 (5 gene) and 100 most upregulated genes in stage 4. The gene sets were used later in the GSEA. For identifying gene set of social-stress perturbation-sensitive genes we used the differentially higher expressed genes after social stress perturbation. GSEA was performed for each cell based on the difference to the mean of log normalized expression values of all cells in the analyzed set as pre-ranked list and 1000 randomizations (2, 3). GSEA on the accumulated UMAPs were plotted using the normalized enrichment scores (NES) of stage 1, stage 4 and of social stress perturbed gene-sets were plotted. (**C**) The distribution of HSC, MPP1 and MPP2 cells having positive NES in the different E-clusters.

3) The animals used in these experiments were quite young for evaluating adult HSCs and in some cases there appear to be "batch effects from the mice- could the authors clarify age / sex had any role in the variation seen?

For all experiments we used miR-221/222-proficient and deficient male mice of closely comparable ages, between 6 and 12 weeks of age. At this age, mice are considered as adult and we have not found any systematic difference originates from the age differences of mice used. However, we acknowledge the possibility, that miR-221/222-defficiency could contribute to the aging of hematopoiesis. Since these problems are fascinating, it needs further investigations in another study.

After we have analyzed more mice during the revision and added the data points to the pool of data that we had before, it become apparent that individual values distribute on a wider range, independently from inter-assay variations.

4) The authors present that the miRNA 221/222 depleted cells fail to reconstitute recipient in serial transplantations, however these HSCs are able to long term reconstitute PB, at much lower levels, but also reconstitute the early hematopoietic progenitor compartment robustly. This, together with the myeloid biased output, are similar to phenotypes of aged stem cells- and thus the loss of miRNA 221/222 could lead to increased activation of the stem cell compartment, leading to age-associated phenotypes. The authors should explore this alternative - some suggestions would be to compare profiles of the HSCs after transplant with aged HSCs (understandable if these cells are not available) look for altered CD150 expression levels in the miRNA 221/222 deficient vs proficient etc…

As noted in reply to the point 3 of the referee, our results certainly suggests additional experiments on miR-221/222-dependent influences on aging of hematopoiesis. Again, these are clearly fascinating problems, but beyond the scope of the current paper.

Minor Issues:

1) The introduction and discussion could benefit from some editing to make them more streamlined to ensure the main points don't get diluted.

As this point was addressed by all three Reviewers we rewrote the title, the abstract and revised the introduction and discussion.

2) Please clarify that cell surface markers used throughout for the isolation of cells- in some places it appear CD34- Flk2- was also included while the text indicates LSK CD150+ CD48- was used.

We apologize that the phenotypic characterization of these cells was not clear in the manuscript. After we put the supplementary methods now in the main text, we hope that our gating strategy become clear.

We used LSK CD150+ CD48- (pools of HSC and MPP1 cells) only for validating single cell miR-221/222 expression in miR-221/222 deficient mice as shown on **Fig 2A** or detecting miR-221/222 expression in bulk sorted early stem and progenitor cells as shown in **Supplementary Fig 4A**. All other analyses and cell sorting experiment were done with LSK CD34-/+ Flk2-/+ dissection of CD48-/+ CD150+/- early progenitor cells as it is shown on **Supplementary Fig 5**.

**Reviewer 3:**

In their manuscript, Dr. Peter K. Jani et al. describe the role of miR-221/222 in HSCs and MPPs, highlighting novel potential targets. Overall, the results are interesting, important and novel, if confirmed. However, there are some issues that need to be addressed, as detailed below:

1) How long does the perturbation of the steady-state hematopoiesis due to "in vivo" social stress last? Is it reversible? This is important to evaluate the relevance of the observation.

We have analyzed the recovery from the social stress-perturbed state to unperturbed state by analyzing the hematopoietic stem and progenitor cell compartments of the bone marrow during time of recovery. As our results show, the recovery took 14-21 days.

The detail of the results now incorporated in the manuscript results and shown on a new **Fig 1A** and **Supplementary Fig1A** of the manuscript.

2) Fig 1A, please specify if the figure refers to WT (miR-221/222 proficient) cells. In the same paragraph, Supplementary Fig.1 is quoted (line 151), but it is a bit confusing to see at this point results on Proficient/Deficient cells, while trying to evaluate what is written in the text referred to proficient cells only. Maybe separate figures would be easier to read. Moreover, I strongly suggest to highlight only significant p-values.

The Reviewer is right. **Fig 1** shows data only from miR-221/222-proficient mice. As discussed in the reply to the previous question, we re-made **Fig 1** and **Supplementary Fig1**. We made attempts to make all points as comprehensive as possible. It is worth noting that some data on **Supplementary Fig 1B** **and C** are presented for better comparison on the same panel but on logical reasons they are discussed at different result points of the manuscript.

3) Line 150: according to Supplementary Fig.1, also MPP4 increase upon social stress. Please correct the text.

The Reviewer is right. We have corrected the text and modified the **Fig 1A** included MPP4 as significantly different population and removed it from **Supplementary Fig1**.

4) Line 160: the authors write that Paired t-test was used to evaluate statistical significance. It is not clear to me how the pairing was made, since the comparison was made between stressed and not stressed mice, not among the same mouse before and after stress. Please explain. In addition, was a normality test applied before choosing ttest? Please specify.

We thank for the careful remark, the applied statistical test was not correct. We changed the statistical calculations from paired t-test to one-way ANOWA with Tukey-post normalization test to evaluate significant differences between the numbers of cells detected at different times after transportation induced social-stress perturbation.

5) Supplementary Table 1 is quoted in different parts of the manuscript; however, it is not clear which tab one has to evaluate. An explanation of the Table and of the different tabs is necessary in my opinion. Importantly, I don't see any reference to where the raw data have been deposited in a public repository.

We agree with the Reviewer, that the table is difficult to comprehend. We have now attempted to clarify it in the text, referring always to the appropriate location (tab) in the Table. All data and the manuscript have been deposited and available from bioRxiv under the following accession number: 10.1101/2023.01.30.526397. The single cell RNA-seq datasets are available through NCBI under the following accession numbers GSE227322, GSE227505, GSE227520.

6) I have some difficulties in understanding the approach of using ActD prior to RNAseq, since there is already an internal control of the experiment, for example the equally "ex vivo/in vitro" treated miR-221/222 proficient cells. However, I realize that it allowed to highlight genes that would have not been picked up with other approaches. It would be helpful if the explanation of the reason for choosing ActD-treated cells was more convincing.

We use ActD to inhibit any transcription in cells from the beginning of their isolation, purification and processing “in vitro”, with the aim to preserve (“freeze”) the transcriptional status, which exists ”in vivo” at the beginning of the experiment.

As suggested by Reviewer 1, we are taking his/her suggested reasonable course of action to remove the section of the paper describing the differential single cell RNAseq analyses in the presence and absence of ActD (**Fig 1B**). As suggested by Reviewer 1, we now just state in the Methods and in the Results that “ActD was used during cell isolation and processing to avoid transcription occurring during cell preparation and processing”.

We hope, that Reviewer 3 also agrees with this action.

7) Supplementary Fig 4A: statistics is missing.

We have inserted the missing statistical analyses.

8) Line 285: MPP3 are also increased.

The Reviewer is right. We have corrected the text accordingly.

9) Lines 340-341: this assertion is too strong.

The quoted sentence in line 340-341 (“The results of our experiments indicate, that this pathway is controlled by the miR 221/222 gene cluster.”) is summarizing our experimental results and our conclusions arising from these results. In miR-221/222-deficient mice in unperturbed hematopoiesis we found reduced number of HSC, increased number of MPPs in the bone marrow, and increased numbers of granulocytes in the periphery compared to miR‑221/222-proficient mice and these processes involve IEG activation similar to social stress perturbation. This indicates that whatever the pathway is or the pathways are in these complex processes, miR‑221/222 have some control, which need future exploration. We believe that our assertion is not too strong.

10) The lack of a functional validation of at least some of the putative mir-221/222 cluster indirect targets (in other words, of the transcriptional changes that occur in the absence of mir-221/222, seen here with RNA sequencing) is a strong limitation.

As the comment 7 of Reviewer 1 raises the problem of target validation we gave our answer to that question which would be very similar to the answer we would give here. We hope Reviewer 3 accepts our arguments/reply given to Reviewer 1. As also replied to Reviewer 1, we have changed the wording of our conclusions, no longer to say that Fos IS a target, but that our differential mRNA expression data SUGGESTS it, as we SUGGEST indirect effects on genes mediated by AP1.

11) Lines 557-559: "we have identified…". The fact that miR221 might play an important role in HSPCs had been suggested in at least two previous papers (Felli N et al. PNAS 2005; Crisafulli L et al., Haematologica 2019). In particular, the differences observed here compared to previous publications should be discussed, with some hypothesis to explain discrepancies. For example, in Felli et al., which the authors quote, the demonstrated miR-221/222 direct target cKit does not appear as differentially expressed in the present manuscript. In Crisafulli et al., the mature form of miR-221 was found to be only minimally expressed in HSCs, and strongly upregulated in MPP1.

We are thankful to the Reviewer for drawing our attention to the results of the two studies. We believe that our findings are rather supported by these two studies. We added the work of Crisafulli et al.(4) to our reference list.

Strikingly, our data on bone marrow derived hematopoietic stem cells of mice show no alteration in kit mRNA expression in miR‑221/222-deficient cells. This is in line with the results of Felli et al, i.e. mRNA expression changes of kit in human cord blood derived HSC and later progenies in absence or presence of human miR‑221 was not detectable. Our analysis did not concentrate on the regulatory role of miR‑221/222 on translational repression; we focused on the effects of the two microRNA on transcriptional changes of target mRNAs.

Crisafulli et al analyzed miRNA expression profiles in LSK CD34^-^CD135^-^ cells (considered as HSC) and in LSK CD34^+^CD135^-^ cells (considered as MPP) after polyI:C injection in mice. In these analyses, they found low level of miR‑221 in HSC and ~570-fold increased expression of miR‑221 in MPPs, while interestingly no changes in miR‑222 level. Since we measured miR‑221/222 expression in single unperturbed (not in social stress perturbed or polyI:C-stressed) LSK CD34^-^CD135^-^CD150^+^CD48^-^ HSCs, LSK CD34^+^CD135^-^CD150^+^CD48^-^ MPP1 and LSK CD34^+^CD135^-^CD150^+^CD48^+^ MPP2 cells, our results are not directly comparable to the findings of Crisafulli et al.

Worth noting is, that stress induced by polyIC might suppress miR‑221 expression in HSC and induce it’s expression in later MPP populations. This would be in line with our conclusion that miR‑221/222 is involved in regulation of stress-responses of hematopoietic progenitors, which might need it’s adapted expression upon stress. Additionally, our analysis on miR-221/222 expression in MPP3 + MPP4 populations (LSK CD150-CD48+) even in unperturbed mice show an elevated expression compared to LSK CD150+CD48- LT-HSC (**Supplementary Fig 4A**), which might be the indication of similar result described by Crisafulli et al. where the miR-221 expression is compared on greater phylogenetic distances.

12) Too many assumptions in the Discussion (which is a bit too long), considering the absence of a validation for all the quoted genes.

Please find our reply written to the comment/question of Reviewer 1 and Reviewer 2, we have entirely rewritten the title, the abstract, revised the introduction and the discussion.

13) The resolution of most figures is not good. In particular, in Fig. 1B gene names are almost impossible to read.

For faster processing the publisher does not require high quality figures for the first submission. Of course, we have all the figures in high resolution, upon request we can provide them. We apologize, that figure **Fig 1B** was difficult to read, in the revised version the panel has been removed from the figure.

14) The manuscript is not particularly fluent and a bit difficult to read; there are some grammar mistakes. In addition, it is a bit confusing the fact that results obtained with miR-221/222 deficient cells appear later in the manuscript. I would rather describe deficient mice first, and then compare the results with those obtained with stressed WT mice, thus changing the structure of the manuscript. This sequence is the one that the authors followed in the Abstract. Moreover: the introduction is too long; the first paragraph of the Results is redundant, everything has been written in the Intro.

Please see our reply to comment 12.

Minor issues:

- There are three acronyms in the title.

We gave the paper a new title.

- Lines 255-267: it is a copy and paste of lines 243-254.

We have deleted the repetition. We thank for the careful remark.

REFERENCES

1.	Errico MC, Felicetti F, Bottero L, Mattia G, Boe A, Felli N, et al. The abrogation of the HOXB7/PBX2 complex induces apoptosis in melanoma through the miR-221&222-c-FOS pathway. Int J Cancer. 2013;133(4):879-92.

2.	Subramanian A, Tamayo P, Mootha VK, Mukherjee S, Ebert BL, Gillette MA, et al. Gene set enrichment analysis: a knowledge-based approach for interpreting genome-wide expression profiles. Proc Natl Acad Sci U S A. 2005;102(43):15545-50.

3.	Mootha VK, Lindgren CM, Eriksson KF, Subramanian A, Sihag S, Lehar J, et al. PGC-1alpha-responsive genes involved in oxidative phosphorylation are coordinately downregulated in human diabetes. Nat Genet. 2003;34(3):267-73.

4.	Crisafulli L, Muggeo S, Uva P, Wang Y, Iwasaki M, Locatelli S, et al. MicroRNA-127-3p controls murine hematopoietic stem cell maintenance by limiting differentiation. Haematologica. 2019;104(9):1744-55.
