# Supplementary material for: The miR-221/222 cluster regulates hematopoietic stem cell quiescence and multipotency by suppressing both Fos/AP-1/IEG pathway activation and stress-like differentiation to granulocytes
Source: PLoS Biol. 2023 Nov 20;21(11):e3002015. doi: 10.1371/journal.pbio.3002015 (PMC10695376; doi:10.1371/journal.pbio.3002015)
Supplement: Attachment — Submitted filename: Response to Reviewers2.docx [file pbio.3002015.s011.docx]

**Reviewer 1 (Mark Ansel)**

The revised manuscript adequately addresses each of my prior concerns. The additional data provided in the response to reviewers document, regarding predicted miR-221/222 target gene expression in wildtype and miR-221/222-deficient HSC and MPP cells, would be useful for all readers. I recommend including it in the manuscript. From the violin plots, it does appear that the predicted targets are slightly shifted in their expression in miR-221/222 deficient cells as predicted. CDF plots would probably make this clearer, but in any case these results help to clarify the direct versus indirect effects of miR-221/222. With regard to Fos, I agree with the authors handling of the discussion of this gene, which may indeed be both a direct AND indirect target in this case. Finally, I am sympathetic to the difficulty of repeating the serial transplantation experiments. Nevertheless, I do recommend that readers treat those results with caution, since they have not been replicated in an independent experiment.

We are thankful to Mark Ansel for his positive and supportive evaluation of our manuscript. According to his suggestion, we have inserted a new Figure (Fig 4) and its caption in the manuscript, as well as a paragraph text in the results (Lines 314-326). With accepting the possibility to publish the peer review history of the manuscript, we would like to grant the readers the possibility to be aware on your concerns on our transplantation results.

**Reviewer 3**

I thank Dr. Peter K. and colleagues for addressing all the issues that I have raised, as well as the ones raised by the other reviewers. The manuscript has substantially improved.

We are thankful to Reviewer 3 for the positive evaluation of our manuscript.
